# Supplementary material for: Evolutionary dynamics of pseudoautosomal region 1 in humans and great apes
Source: Genome Biol. 2022 Oct 17;23:215. doi: 10.1186/s13059-022-02784-x (PMC9575207; doi:10.1186/s13059-022-02784-x)
Supplement: Supplementary file 2 — Additional file 2. Supplementary figures and legends. [file 13059_2022_2784_MOESM2_ESM.docx]

# Additional file 2

## Supplementary figures


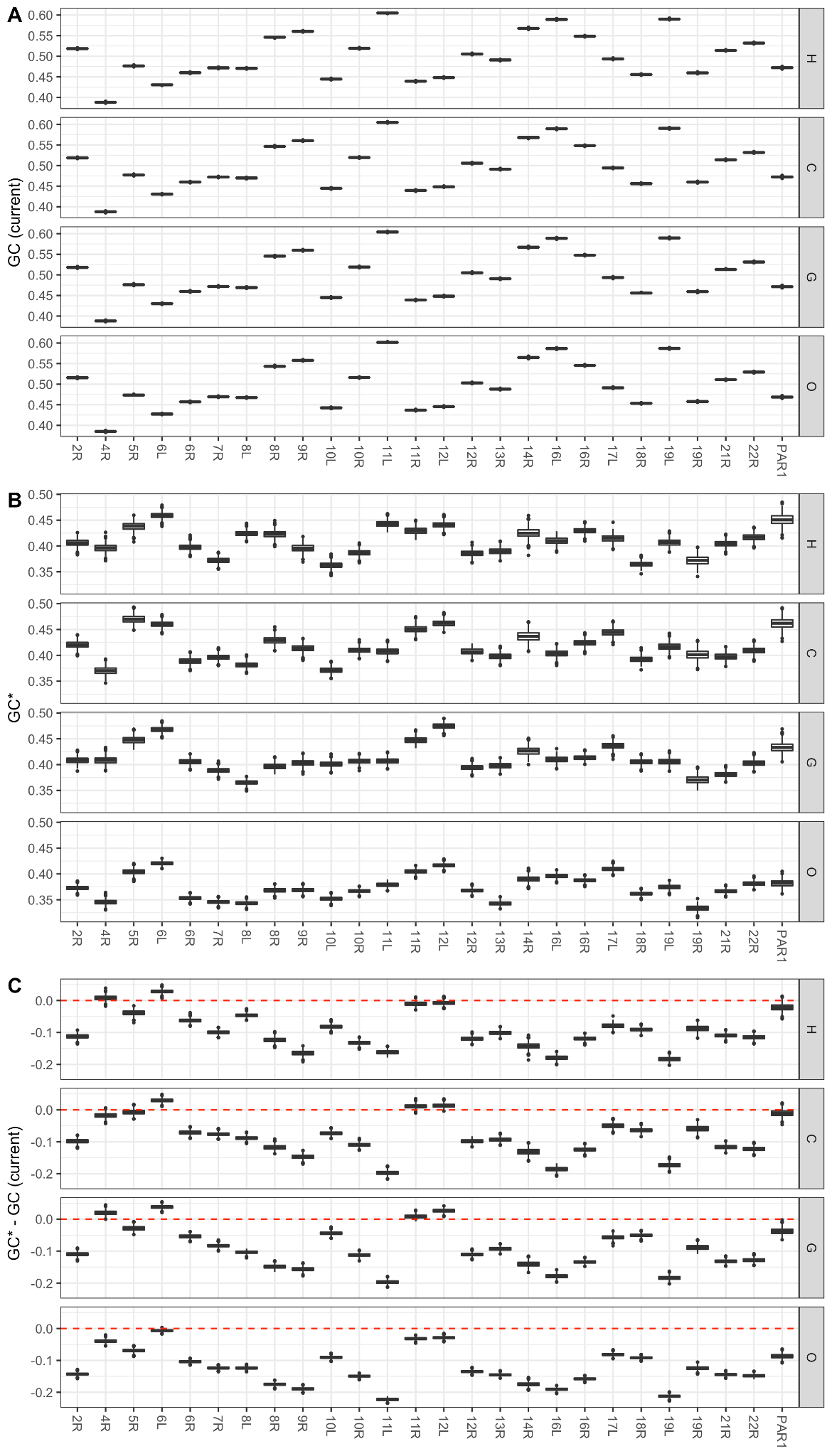


Figure S1. Bootstrap intervals for **A.** current GC in telomeres of the human (H), chimpanzee (C), gorilla (G) and orangutan (O) genome, **B.** equilibrium GC*, and **C.** difference between GC* and current GC. The red dashed lines indicate the expectation under nucleotide composition equilibrium. Each bootstrap interval was estimated from 1,000 bootstrap samples.


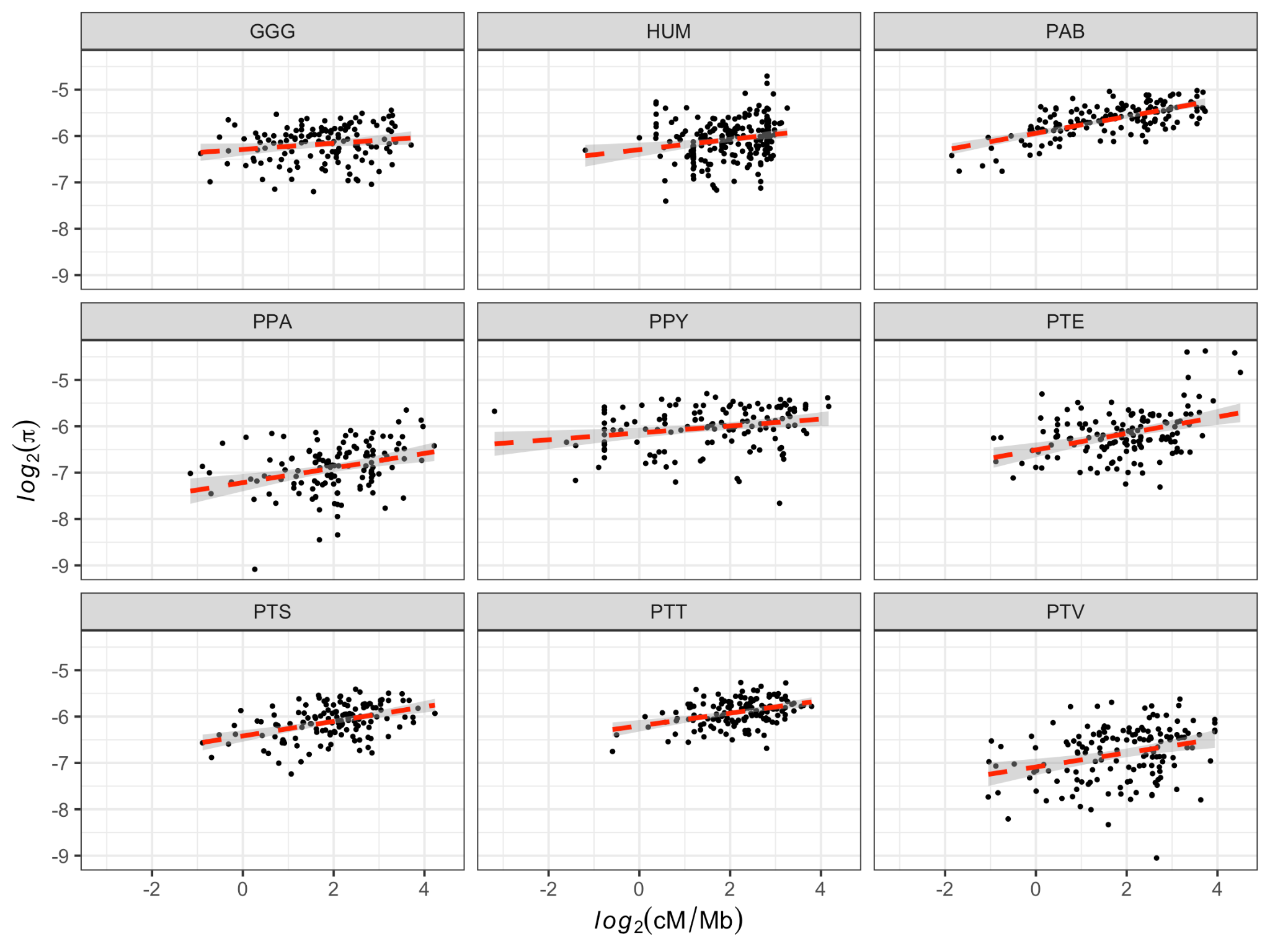


Figure S2. Correlation of recombination rate and nucleotide diversity, measured as 𝜋, for 10 kb windows of PAR1 in humans (HUM) and eight subspecies of great apes (PTE = *P. troglodytes ellioti*; PTS = *P. troglodytes schweinfurthii*; PTT = *P. troglodytes troglodytes*; PTV = *P. troglodytes verus*; PPA = *P. paniscus*; GGG = *G. gorilla gorilla*; PAB = *P. abelii*; PPY = *P. pygmaeus*). We only consider 10 kb regions with more than 2,500 callable sites.
