# Supplementary material for: Evolutionary dynamics of pseudoautosomal region 1 in humans and great apes
Source: Genome Biol. 2022 Oct 17;23:215. doi: 10.1186/s13059-022-02784-x (PMC9575207; doi:10.1186/s13059-022-02784-x)
Supplement: Supplementary file 3 — Additional file 3. Analysis of sexual antagonism in PAR1 of humans a great apes. [file 13059_2022_2784_MOESM3_ESM.docx]

# Additional file 3

## No evidence for sexually antagonistic alleles in PAR1

As regions closely linked to the pseudoautosomal boundary can potentially maintain polymorphisms for sexually antagonistic (SA) alleles under a wider parameter range than other genome regions, we looked for differences in sex-specific allele frequencies. In Figure S3A, we show absolute frequency differences between females and males for all segregating sites and species. Frequency differences for the human YRI population reach to about ~25%, and up to 100% for great ape populations. However, when applying Fisher’s exact tests to assess the significance of frequency differences, followed by Bonferroni's correction for multiple testing, we found no significant frequency differences in any of the populations (Figure S3B). Due to the small sample sizes for non-human populations, the significance threshold is unreachable for most of these populations, as demonstrated by a SNP at position 180,684 for the *P. paniscus* population. Although this SNP is fixed for different alleles in females and males, the frequency difference is non-significant after *p*-value correction. In the human YRI population, the SNP that is closest to a significant frequency difference is located at position 1,322,855 (rs146912994) in the intron of the *CSF2RA* gene. The T allele of this SNP segregates at 15.69% frequency in males, while females are fixed for the C allele. Furthermore, this SNP is not present in Eurasian populations [(82)](https://paperpile.com/c/CBQjyh/WpTTz). However, according to the dbSNP database (<https://www.ncbi.nlm.nih.gov/>), there are currently no phenotypes associated with this SNP that would imply sexually antagonistic evolution.

Since PAR1 segregates for a large number of sites, we were able to test whether frequency differences increase with proximity to the pseudoautosomal boundary, as would be expected in the presence of SA selection [(6)](https://paperpile.com/c/CBQjyh/Y1cpm). We separated segregating variants into 20 non-overlapping windows, each approximately 1 cM in genetic length, and calculated window-based average differences between female and male allele frequencies. We find no evidence for such a SA gradient (Figure S3C), nor generally any significant differences between sex-specific allele frequencies when applying Fisher’s exact test to the sums of female and male allele counts in each window (Figure S3D).


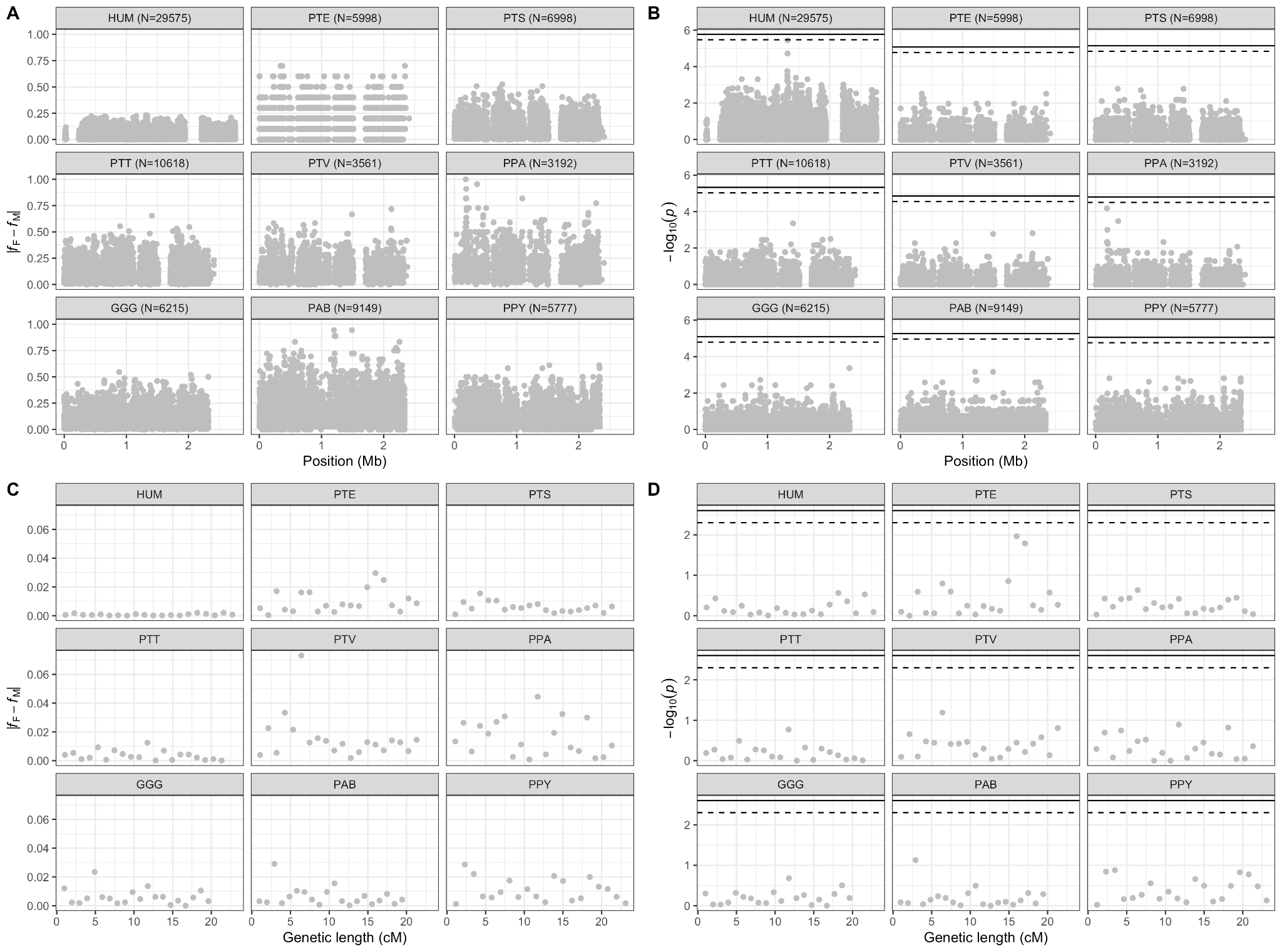


Figure S3. **A.** Absolute differences between female (*f*_F_) and male (*f*_M_) allele frequencies for sites segregating in humans (HUM) and eight subspecies of great apes (PTE = P. troglodytes ellioti; PTS = P. troglodytes schweinfurthii; PTT = P. troglodytes troglodytes; PTV = P. troglodytes verus; PPA = P. paniscus; GGG = G. gorilla gorilla; PAB = P. abelii; PPY = P. pygmaeus). Numbers of polymorphic sites plotted in each panel are presented in the parentheses. **B.** *p*-values of Fisher’s exact tests for between-sex allele frequency differences. **C.** Window-based absolute differences between female and male allele frequencies for sites segregating in 20 windows of approximately 1 cM in genetic length in humans and eight subspecies of great apes. **D.** Window-based *p*-values of Fisher’s exact tests for average between-sex allele frequency differences. The solid and dashed horizontal lines in panels **B** and **D** represent the significance thresholds at *p* < 0.05 and *p* < 0.1, respectively, after Bonferroni’s correction for multiple testing.
