# Supplementary material for: Evolutionary dynamics of pseudoautosomal region 1 in humans and great apes
Source: Genome Biol. 2022 Oct 17;23:215. doi: 10.1186/s13059-022-02784-x (PMC9575207; doi:10.1186/s13059-022-02784-x)
Supplement: Supplementary file 4 — Additional file 4. Analysis of factors that ensure recombination in PAR1. [file 13059_2022_2784_MOESM4_ESM.docx]

# Additional file 4

## Assurance of recombination in PAR1

A recombination event in the pseudoautosomal region during male gametogenesis is crucial for proper sex chromosome segregation and male fertility. In mice, ensuring PAR recombination has been shown to be associated with a unique chromatin structure of the PAR, mediated by repetitive DNA [[55]](https://paperpile.com/c/oRuVnx/yU0bs). We therefore sought to characterise repeat elements of the human PAR1, using autosomal telomeres as a reference point. Specifically, we used the Tandem Repeats Finder program [[97]](https://paperpile.com/c/oRuVnx/LSeQI) to analyse PAR1 and the terminal 3 Mb regions of autosomes, to obtain basic statistics about their repeat content (Figure S4). The largest number of repeat elements was found in PAR1. Furthermore, PAR1 elements usually contained more copies of the consensus pattern, compared with the autosomal regions. In total, almost 25% of PAR1 is composed of repeat sequences - approximately double the proportion of the most repeat-rich autosomal telomeres. Additionally, PAR1 repeats contain the highest proportions of mismatches and indels between adjacent copies. Together, these results imply an unusual repeat structure in the human PAR1, which may aid in recombination assurance, as observed in mice.

Another important determinant of recombination is binding of the recombination motif-recognition protein PRDM9 that mediates meiotic double-strand break (DSB) formation, and was shown to operate in the human PAR1 [[56]](https://paperpile.com/c/oRuVnx/z5OD3). We explored the sequence evolution of PAR1 regions within DSB hotspots inferred for individual human males [[56]](https://paperpile.com/c/oRuVnx/z5OD3). Specifically, we divided sites of the great ape PAR1 alignment into those falling outside (217,856/239,799; 90.85%) and inside (21,943/239,799; 9.15%) of human DSB hotspots, and ran the phyloFit program to determine divergence rates for the two classes of sites (Table S2). We observe higher divergence of sites within DSB hotspots for the human PAR1, compared with non-hotspot regions, as expected due to the higher rate of sequence evolution within these regions [[56]](https://paperpile.com/c/oRuVnx/z5OD3). Interestingly, the same pattern also holds for all other great ape species. Given that the DSB regions were characterized in human males, the fold-increase in divergence is greatest for the human sequence (1.32), as expected, and ranges from 1.14 to 1.3 in the other great ape species. Additionally, substitution spectra for DSB regions show an excess of C→G transversions and a paucity of CpG→TpG transitions compared to non-DSB regions (Figure S5), indicating a stronger mutagenic effect of male recombination, as well as stronger gBGC within these regions, across all great ape species.


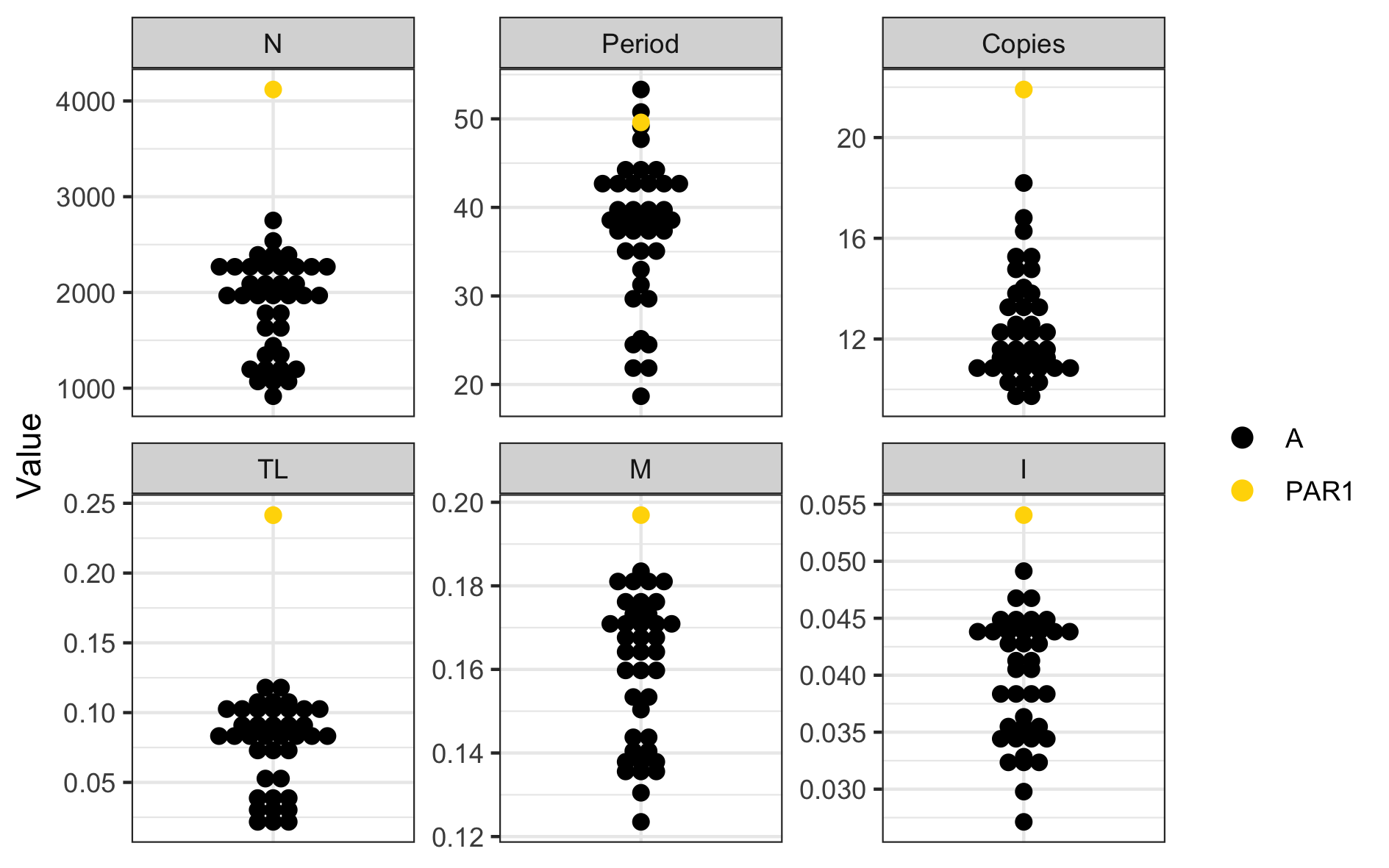


Figure S4. Repeat element statistics for human autosomal (A) telomeres and PAR1. The plotted statistics are as follows: N = the total number of detected repeat elements, Period = the mean period size of the detected repeat elements, Copies = the mean number of copies of the repeating pattern in detected repeat elements, TL = proportion of the total length of the telomere that is covered by repeat elements, M = mean proportion of mismatches between copies of the detected repeat elements, I = mean proportion of indels between copies of the detected repeat elements. In total, we plot 40 points in each panel corresponding to PAR1 and 39 autosomal telomeres (the heterochromatic p-arm telomeres of chromosomes 13, 14, 15, 21 and 22 were excluded from the analysis due to their poor sequence assembly).


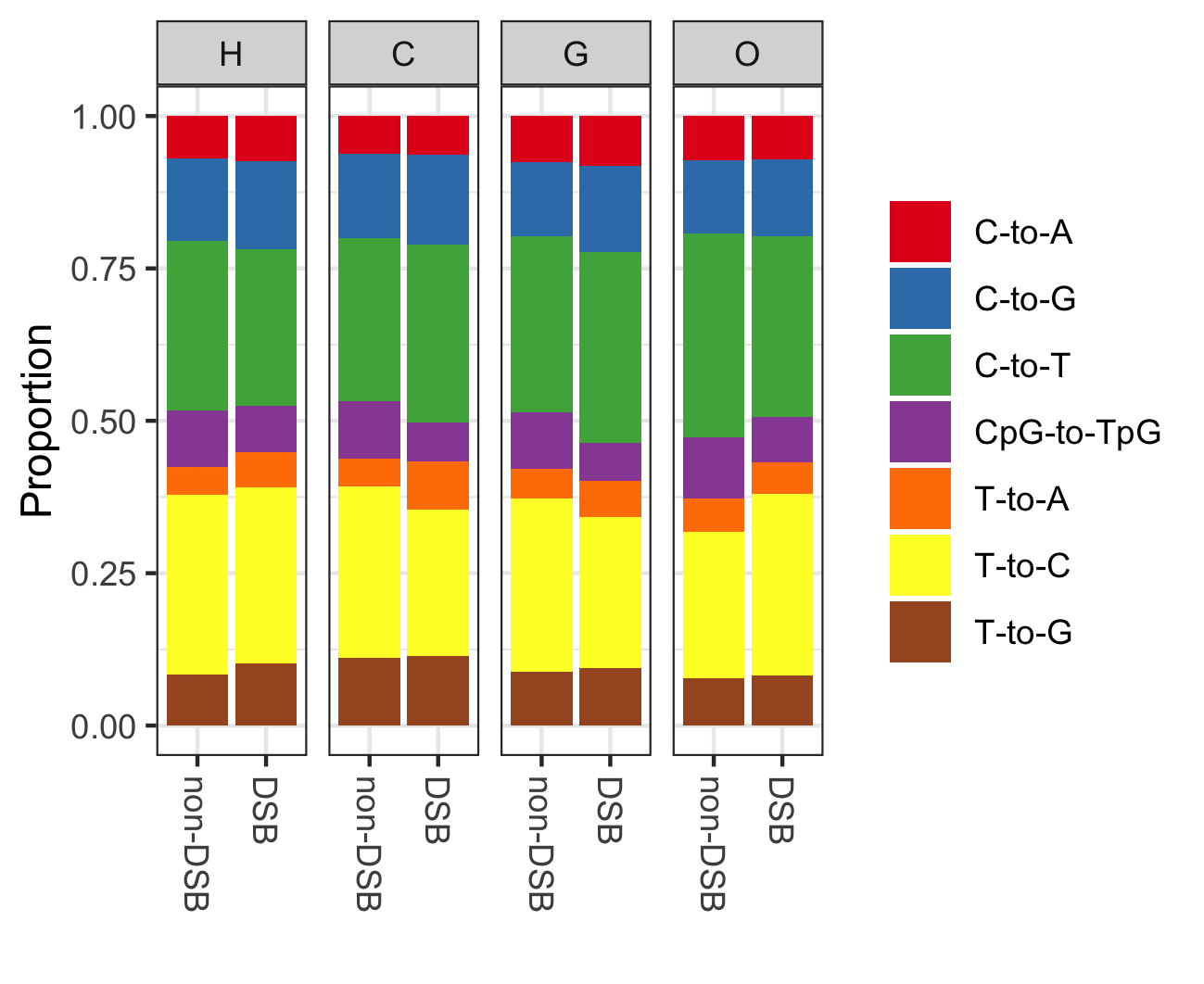


Figure S5. Substitution spectra for the human (H), chimpanzee (C), gorilla (G) and orangutan (O) PAR1 of regions outside and inside of double-strand break hotspots (non-DSB and DSB, respectively).

Table S2. Divergence estimates based on phyloFit estimation and divergence times from [[62]](https://paperpile.com/c/oRuVnx/Vesxq) for PAR1 regions outside and inside double-strand break (DSB) hotspots as determined by [[56]](https://paperpile.com/c/oRuVnx/z5OD3).

|  | Outside DSB hotspots | | Inside DSB hotspots | |
| --- | --- | --- | --- | --- |
|  | Total divergence | Per site and year divergence | Total divergence | Per site and year divergence |
| Human | 0.0102 | 0.9373×10^-9^ | 0.0135 | 1.2453×10^-9^ |
| Chimpanzee | 0.0117 | 1.0724×10^-9^ | 0.0152 | 1.3938×10^-9^ |
| HC ancestor | 0.0037 | 1.7583×10^-9^ | 0.0038 | 1.8105×10^-9^ |
| Gorilla | 0.0141 | 1.0859×10^-9^ | 0.0162 | 1.2523×10^-9^ |
| HCG ancestor | 0.0164 | 1.5087×10^-9^ | 0.0185 | 1.7001×10^-9^ |
| Orangutan | 0.0275 | 1.1512×10^-9^ | 0.0326 | 1.3659×10^-9^ |
