# Supplementary material for: Evolutionary dynamics of pseudoautosomal region 1 in humans and great apes
Source: Genome Biol. 2022 Oct 17;23:215. doi: 10.1186/s13059-022-02784-x (PMC9575207; doi:10.1186/s13059-022-02784-x)
Supplement: Supplementary file 5 — Additional file 5. Review history. [file 13059_2022_2784_MOESM5_ESM.docx]

Review History

**First round of review**

**Reviewer 1**

**Are you able to assess all statistics in the manuscript, including the appropriateness of statistical tests used?** Yes, and I have assessed the statistics in my report.

**Comments to author:**

In their manuscript entitled "Evolutionary dynamics of pseudoautosomal region 1 in humans and great apes" Bergman and Schierup analyze the the region PAR1, a region on the human sex chromosomes, which is responsible to ensure proper segregation during male meiosis.

I have some questions and points of critique:


- the authors report rates per sit and year (PY). What times of the last common ancestors (internal nodes in the phylogeny) have been used and where have they been taken from? To circumvent circular arguments these numbers have to come from analyses not using sequence data, i.e. from the fossil record.

- In figure 2 a boxplot of divergence rates is shown. For "PAR1" there are four data points. In my opinion these 4 data points are independent from each other and a box plot is not appropriate. Similar for the data for autosomes. Each branch evolves independent from the others. It is also possible that the autosomes evolve differently (e.g. telocentric chromosomes could evolve faster or slower). See also Fig 5 for trends.

- Similarly in figure 3 the data for GC and GC* should not be accumulated. From figure 4 it actually becomes clear that for 3 of the branches GC and GC* are actually quite close together. Which branches are this? I would find an plot of GC* vs GC more appropriate.

- Autosomal telomeres have been chosen for comparison. However even they are not evolving homogeneously and show difference among them - see Figure 5. How do non-telomeric regions evolve in comparison? Would they serve as well?

- Along the same line: also the X and Y chromosomes have another telomere - wat rates would be measured there. What about region on X and Y away from the telomeres?

- PAR1 is situated on the sex chromosomes which spend different amounts of time in the male and female germline. How does effect the current analysis? Could the difference in the total divergence (see Table 1) be attributed to this effect?

- Does the alignment length allows to measure the rates so accurate that one can say that the difference of GC and GC* is actually significant?

- What is the error bar on these rates? A bootstrap analysis should be possible.

- Figure 1 should also include the outgroup species.

- How is GC* computed?

- the y-axis in Figure 6C is not labeled.

- As such shown analysis is not easy to reproduce. Would it be possible to have access to computational notebooks (using R-markdown or something similar)?

**Reviewer 2**

**Are you able to assess all statistics in the manuscript, including the appropriateness of statistical tests used?** Yes, and I have assessed the statistics in my report.

**Comments to author:**

This ms by Bergman and Schierup reports a comprehensive analysis of the patterns of divergence and diversity in the pseudoautosomal region (PAR1) of great apes, including modern and extinct humans, using publicly available data. This analysis allows them to draw conclusion about evolutionary forces such as recombination, mutation and biased gene conversion acting on PAR1 (using autosomal telomeres for comparison). They also looked for evidence of sexually-antagonistic selection in PAR1 but could not find any. This result is not in agreement with a recent study in humans by another group; possible reasons for discrepancy are discussed. They also found that PAR1 has not been introgressed from modern humans to Neanderthals and Denisovans. This is interesting because a recent study has shown that the modern human Y has introgressed in Neanderthals and Denisovans.
There are a lot of work and results reported in this long ms. I found the analyses well done. The most interesting and novel part is, I think, the part on PAR1 having not introgressed from modern humans to Neanderthals and Denisovans. It is however a small part of the ms at present and is not properly discussed.

I have few comments mostly about the discussion, which I found was very short given all the data presented:
1. At present the discussion is focused on discrepancy with Monteiro et al. paper and this is fine to have such discussion, but I think it would be useful to have a more general discussion about sexually antagonistic selection in primates. Including this paper on in the Discussion (and maybe introduction?) could help:
Shearn R, Wright AE, Mousset S, Régis C, Penel S, Lemaitre JF, Douay G, Crouau-Roy B, Lecompte E, Marais GA. Evolutionary stasis of the pseudoautosomal boundary in strepsirrhine primates. Elife. 2020 Nov 18;9:e63650. doi: 10.7554/eLife.63650. PMID: 33205751; PMCID: PMC7717902.
2. I also found the discussion on the introgression analysis very short. What are your hypotheses to explain that PAR1 had not introgressed along with the rest of the Y chromosome?

The part on the sexually-antagonistic selection is interesting but, as the authors acknowledged themselves, their negative results may be due to lack of statistical power or the use of a single human population. More work (using new data and possibly other methodologies) is needed as they rightly say. No strong conclusion (even negative) can be drawn on this part. The ms could be published in Genome Biology, provided that it is reframed, focused on the introgression part and greatly shortened.

**Authors Response**

**Response to Reviewer 1**

Reviewer #1:

In their manuscript entitled "Evolutionary dynamics of pseudoautosomal region 1 in humans and great apes" Bergman and Schierup analyze the the region PAR1, a region on the human sex chromosomes, which is responsible to ensure proper segregation during male meiosis.

I have some questions and points of critique:

- the authors report rates per sit and year (PY). What times of the last common ancestors (internal nodes in the phylogeny) have been used and where have they been taken from? To circumvent circular arguments these numbers have to come from analyses not using sequence data, i.e. from the fossil record.

**Response: The times of the last common ancestors (LCA) have been independently estimated in Besenbacher et al. (2019; https://doi.org/10.1038/s41559-018-0778-x). These authors produced species-specific yearly mutation rates of all great ape species (estimated using de novo sequencing of parent-offspring trios). Using these mutation rate estimates and autosomal-based divergence rates from Scally et al. (2012; https://doi.org/10.1038/nature10842 ), they inferred times to the LCA. Furthemore, the authors showed that LCA times correspond well to the fossil record (Figure 3 in Besenbacher et al., 2019). Given the independence of LCA time estimates and their correspondence to the fossil record, we trust that we have alleviated circular reasoning. We have now made this clearer at the end of the subsection “Divergence and substitution spectra estimation” of the “Methods” section.**

- In figure 2 a boxplot of divergence rates is shown. For "PAR1" there are four data points. In my opinion these 4 data points are independent from each other and a box plot is not appropriate. Similar for the data for autosomes. Each branch evolves independent from the others. It is also possible that the autosomes evolve differently (e.g. telocentric chromosomes could evolve faster or slower). See also Fig 5 for trends.

**Response: We agree with this assessment - in order to achieve more clarity, we have redone Figure 2 (now Figure 2A in main text) into a violin distribution plot with individual points, separated by species, with the PAR1 region highlighted in yellow. We hope that this provides a more appropriate representation of these data.**

- Similarly in figure 3 the data for GC and GC* should not be accumulated. From figure 4 it actually becomes clear that for 3 of the branches GC and GC* are actually quite close together. Which branches are this? I would find an plot of GC* vs GC more appropriate.

**Response: We have reworked Figure 3 (now Figures 2B and 2C in main text) to display the full distribution of the parameters to better accompany the results discussed in the main text. Regarding the branches - human, chimpanzee and gorilla PAR1 branches cluster more closely than the outlier orangutan branch, as is expected due to their evolutionary relationships.**

- Autosomal telomeres have been chosen for comparison. However even they are not evolving homogeneously and show difference among them - see Figure 5. How do non-telomeric regions evolve in comparison? Would they serve as well?

**Response: Autosomal telomeres were chosen due to their similarity with the PAR1 region, both with respect to high recombination rates as well as their positioning on the chromosomes. We expect these two features to be crucial for the evolution of telomeric sequences and argue that they provide a fair comparison to the PAR1 region. We have not used non-telomeric regions as a point of comparison, as the criteria for choosing such regions in order to provide a fair comparison to PAR1 are not obvious to us (given the variation of non-telomeric regions regions with respect to recombination and distance from telomeres). Furthermore, an additional analysis of non-telomeric regions would likely make the analysis less clear-cut, and the manuscript longer and more complex. Even though such a comparison would be interesting for a future study (as well as with non-telomeric sex chromosome regions), we argue that it is outside of the scope of the current study.**

- Along the same line: also the X and Y chromosomes have another telomere - wat rates would be measured there. What about region on X and Y away from the telomeres?

**Response: The reason we did not analyze the other telomere of the sex chromosomes is because it is not syntenic among species - i.e., PAR2 is a human-specific region that is not present in other great apes, and therefore could not be analyzed in the comparative framework that is the basis of all analyses in the manuscript. Regarding X/Y regions away from the telomeres, they have specificities that are due to different mutation rates in male and female gametes, as well as roles in sex determination and sex-specific gene expression. Due to these reasons, an analysis of non-telomeric sex chromosome regions would require a careful and directed analysis, that takes into account these specificities. Such an analysis merits a stand-alone investigation and if added to the current manuscript, would detract from the analyses of telomeric regions which are the focus of our study.**

- PAR1 is situated on the sex chromosomes which spend different amounts of time in the male and female germline. How does effect the current analysis? Could the difference in the total divergence (see Table 1) be attributed to this effect?

**Response: We do not expect PAR1 divergence to be affected by the difference in time spent in male and female germlines, as this is a characteristic of non-PAR regions of the sex chromosomes only. This difference would only be possible in case of low recombination in PAR1, which would allow it to obtain strong linkage to an X or Y chromosome, and develop non-PAR sex-specific characteristics. However, given that PAR1 recombines between X and Y in the male germline (almost) every generation, this scenario is very unlikely, and would require extremely strong sexually selective forces (which we do not detect in our analysis of sexual antagonism in the PAR). Furthermore, since both the X and Y have the PAR1 region, the number of these regions is equal between the sexes, and therefore also the amount of time they spend in each of the sexes is equal. Given these reasons, the difference in divergence is therefore unlikely to be a sex-specific effect.**

- Does the alignment length allows to measure the rates so accurate that one can say that the difference of GC and GC* is actually significant?

- What is the error bar on these rates? A bootstrap analysis should be possible.

**Response: This is a very good point. In response, we have performed a bootstrap analysis of current GC and GC*, presented in the following figure. The bootstrap intervals are fairly small for current GC (Figure S1A) - a simple ratio of sites fixed for GC over the total number of sites in the alignment. On the other hand, calculation of the GC* parameter involves a ratio of substitution rates, i.e. it involves sites that are not fixed for the same nucleotide in all species, which are much rarer than fixed sites, thus making the bootstrap intervals for GC* somewhat wider (Figure S1B). Despite this, the variation in these parameters between the telomeres is visible and the bootstrap intervals do not overlap for most of the pairwise telomere comparisons. This is likely due to the fact that these parameters are estimated using the whole sequence for each telomere, where the total amount of nucleotide sites exceeds 100,000, thus ensuring fairly accurate parameter estimation. We have now added Figure S1 as Supplementary material of the manuscript.**

- Figure 1 should also include the outgroup species.

**Response: We have now inserted the outgroup into Figure 1.**

- How is GC* computed?

**Response: GC* is computed as the ratio of the AT-to-GC divergence rate to the full rate (i.e., the sum of AT-to-GC and GC-to-AT rates). These rates are in turn inferred using the phyloFit program. As input, phyloFit uses a multiple-species alignment with reconstructed ancestral states (which we produced using the Progressive Cactus aligner) to produce a node-specific rate matrix consisting of rates between every pair of nucleotides. We now make this more clear in the manuscript (end of the subsection “Divergence and substitution spectra estimation” of the “Methods” section).**

- the y-axis in Figure 6C is not labeled.

**Response: We have now labeled the axis (Please note that, due to restructuring of the manuscript, this is now Figure 5C).**

- As such shown analysis is not easy to reproduce. Would it be possible to have access to computational notebooks (using R-markdown or something similar)?

**Response: We certainly support full reproducibility. All the scripts and data needed to reproduce the analysis are now available in a dedicated github repository (https://github.com/jbergman/par1EvoDynamics).**

**Response to Reviewer 2**

Reviewer #2: This ms by Bergman and Schierup reports a comprehensive analysis of the patterns of divergence and diversity in the pseudoautosomal region (PAR1) of great apes, including modern and extinct humans, using publicly available data. This analysis allows them to draw conclusion about evolutionary forces such as recombination, mutation and biased gene conversion acting on PAR1 (using autosomal telomeres for comparison). They also looked for evidence of sexually-antagonistic selection in PAR1 but could not find any. This result is not in agreement with a recent study in humans by another group; possible reasons for discrepancy are discussed. They also found that PAR1 has not been introgressed from modern humans to Neanderthals and Denisovans. This is interesting because a recent study has shown that the modern human Y has introgressed in Neanderthals and Denisovans.

There are a lot of work and results reported in this long ms. I found the analyses well done. The most interesting and novel part is, I think, the part on PAR1 having not introgressed from modern humans to Neanderthals and Denisovans. It is however a small part of the ms at present and is not properly discussed.

I have few comments mostly about the discussion, which I found was very short given all the data presented:

1. At present the discussion is focused on discrepancy with Monteiro et al. paper and this is fine to have such discussion, but I think it would be useful to have a more general discussion about sexually antagonistic selection in primates. Including this paper on in the Discussion (and maybe introduction?) could help:

Shearn R, Wright AE, Mousset S, Régis C, Penel S, Lemaitre JF, Douay G, Crouau-Roy B, Lecompte E, Marais GA. Evolutionary stasis of the pseudoautosomal boundary in strepsirrhine primates. Elife. 2020 Nov 18;9:e63650. doi: 10.7554/eLife.63650. PMID: 33205751; PMCID: PMC7717902.

**Response: We thank the reviewer for bringing this paper to our attention. We have now referenced it both in the Introduction and Discussion sections of the manuscript.**

2. I also found the discussion on the introgression analysis very short. What are your hypotheses to explain that PAR1 had not introgressed along with the rest of the Y chromosome?

**Response: A possible hypothesis for human PAR1 not introgressing with the rest of the Y chromosome is its decoupling from the rest of the human Y chromosome due to high PAR1 recombination, followed by loss from the neanderthal lineage due to directional selection (against human PAR1) or simply through genetic drift. We have now added possible hypotheses about this observation in the Discussion section of the manuscript.**

The part on the sexually-antagonistic selection is interesting but, as the authors acknowledged themselves, their negative results may be due to lack of statistical power or the use of a single human population. More work (using new data and possibly other methodologies) is needed as they rightly say. No strong conclusion (even negative) can be drawn on this part.

Re**sponse: We agree, and have decided to transfer that part of the analysis from the main text into an Additional file, and we now refer to these results in the Discussion section.**

The ms could be published in Genome Biology, provided that it is reframed, focused on the introgression part and greatly shortened.

**Response: We have restructured the manuscript such that it gives more emphasis to the introgression part, and have reallocated certain parts of the manuscript into Additional files (e.g. the part about sexually antagonistic selection and the repeat content analysis).**

**Second round of review**

**Reviewer 1**

All my comments have been addressed appropriately. I have no further comments.

**Reviewer 2**

The authors have made some effort to address my comments, and the ms has been improved. I am, however, still doubtful about the priority for GB to publish this ms.
